# Azygos Vein Pseudoaneurysm Secondary to Blunt Chest Trauma: A Case Report

**DOI:** 10.7759/cureus.71243

**Published:** 2024-10-11

**Authors:** Creighton Kellum, Lou Smith, Bracken Burns

**Affiliations:** 1 Department of Surgery, East Tennessee State University Quillen College of Medicine, Johnson City, USA; 2 Department of Surgery, University of Tennessee Medical Center, Knoxville, USA

**Keywords:** azygos vein pseudoaneurysm, blunt trauma chest, isolated injury, trauma ct imaging, traumatic pseudoaneurysm, traumatic vascular injury

## Abstract

Azygos vein aneurysms (AVAs) and azygos vein pseudoaneurysms (AVPs) are typically asymptomatic, discovered incidentally, and most often result from idiopathic and congenital factors or conditions that increase central venous pressure. Trauma-related azygos injuries are particularly rare, with a paucity of reported cases resulting from blunt mechanisms. There is no standardized therapeutic approach to traumatic AVA/AVPs, with observation, endovascular, thoracoscopic, and open surgical interventions all represented in the current literature. We present a case of a 44-year-old female who developed a pseudoaneurysm following a motor vehicle collision. The absence of aneurysmal findings in prior imaging obtained for medical purposes substantiated the traumatic pathogenesis of this pseudoaneurysm. Follow-up post-trauma imaging showed complete resolution of the pseudoaneurysm. Treatment approaches vary due to their rarity and diversity, but this case supports conservative management for small, uncomplicated AVA/AVPs.

## Introduction

Azygos vein aneurysms (AVAs) are rare findings within the posterior mediastinum, usually discovered incidentally in imaging studies [1,2]. These anomalies are generally categorized as idiopathic, secondary to conditions with elevated central venous pressure, or related to congenital anomalies [2,3]. A small subset of azygos vein pathologies, known as azygos vein pseudoaneurysms (AVPs), has been associated with blunt chest trauma, forming secondary to vascular wall tears as opposed to true aneurysmal dilation [4,5]. Most azygos vein injuries resulting from blunt trauma occur alongside other severe injuries, rarely appearing as isolated findings [6]. Though significant chest trauma was traditionally thought to be necessary to produce an AVP, recent cases have shown their formation from seemingly trivial mechanisms [6,7]. While AVAs and AVPs are often asymptomatic, they can lead to serious complications, including rupture, compression of adjacent structures such as the right main bronchus or superior vena cava, and thrombosis leading to pulmonary embolism [2,8,9]. Due to their rarity and the absence of a standardized approach to management, strong guidelines do not currently exist and approaches in the literature vary considerably from conservative surveillance to endovascular procedures, including stenting and embolization to thoracoscopic resection and open thoracotomy [4,5,10,11].

This article was previously presented as a poster presentation at the 2024 Southeastern Surgical Congress Meeting on February 4th, 2024.

## Case presentation

**Figure 1:**
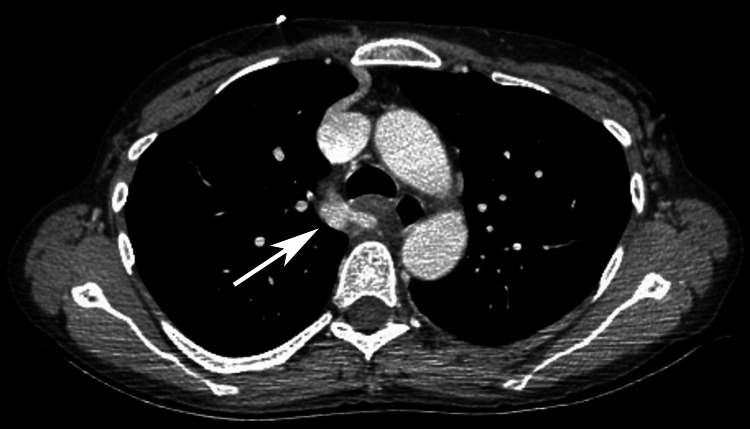
CT of the chest with IV contrast on the day of injury showing azygos vein pseudoaneurysm.

**Figure 2:**
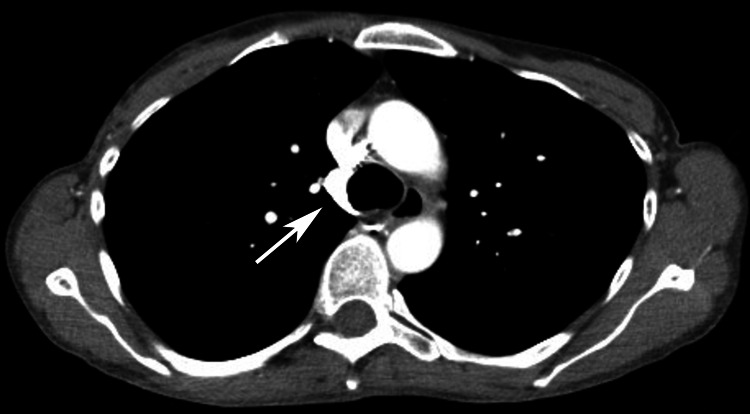
CT of the chest with IV contrast obtained for unrelated reasons one year prior to injury showing no evidence of pseudoaneurysm.

A 44-year-old female with a history of mitral valve prolapse and atrial fibrillation presented as an unrestrained driver in a single-car motor vehicle crash. Initial evaluation revealed the presence of active atrial fibrillation with rapid ventricular response (afib-RVR), which was treated with amiodarone and metoprolol. Her physical exam was notable only for a chin laceration. Given the mechanism of injury and concern for occult injury, a CT scan of the chest, abdomen, and pelvis was obtained revealing a 1.4 x 1 cm transaxial and 1.7 cm craniocaudal AVP with adjacent mediastinal fat stranding (Figure 1). There was no hemothorax, active extravasation, or thrombus within the AVP. No other thoracic or abdominopelvic injuries were identified. A prior chest CT from one year earlier, performed during hospitalization for afib-RVR, showed no evidence of an aneurysm or pseudoaneurysm, substantiating trauma as the etiology (Figure 2).

**Figure 3:**
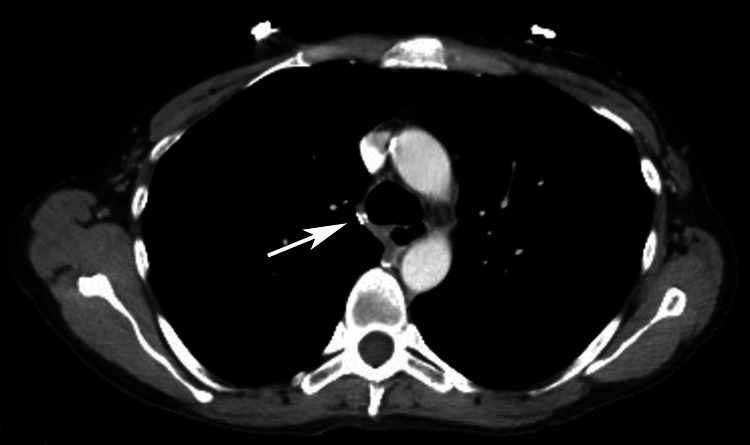
Follow-up CT of the chest with IV contrast eight months post injury showing no evidence of pseudoaneurysm.

Cardiothoracic surgery was consulted, and they determined immediate risk of rupture or thromboembolism was low. Further, they recommended that observation with follow-up imaging was a reasonable approach for this specific injury. A subsequent chest radiograph during her hospitalization showed no expansion or rupture of the AVP. The patient remained asymptomatic, and her atrial fibrillation was controlled. She was discharged home after 24-hour observation, with plans for clinic follow-up and repeat CT imaging if she remained asymptomatic. The patient was lost to follow-up but was readmitted eight months later for afib-RVR. A new chest CT scan revealed no evidence of the previously noted pseudoaneurysm, suggesting spontaneous resolution without intervention (Figure 3).

## Discussion

AVAs and AVPs are rare conditions, predominantly discussed in case reports. There are three main causes thought to contribute to aneurysmal azygos veins: idiopathic/congenital etiologies, pressure or volume overload, and trauma [2,12]. Most reported cases are asymptomatic and discovered incidentally on chest radiography or CT. However, both symptomatic and asymptomatic presentations have been documented for all etiologies [7-9].

Wall et al. described the largest trauma case series of 21 azygos vein injuries, all caused by penetrating mechanisms [13]. Blunt chest trauma as an etiology is exceedingly rare, with limited documented cases [3,5,7,9]. To our knowledge, no previous cases have reported pre-injury imaging confirming the absence of pseudoaneurysm. This report underscores the possibility of AVP formation due to blunt trauma, confirmed by the lack of prior aneurysmal findings on imaging and the presence of peri-injury fat stranding.

While idiopathic true AVAs are often isolated findings, some authors, including Papadomanolakis et al., whose series includes traumatic pseudoaneurysms discovered at autopsy, suggest AVPs are most frequently associated with multiple other injuries such as splenic and renal lacerations, lung injuries, rib fractures, or vertebral fractures [5,6,13]. This suggests that severe mechanisms with extreme forces are required for AVP formation following blunt trauma. However, our report supports the findings by Juraszyński et al., who suggested that AVPs can occur even when overall injury severity is low [7]. Our case demonstrates a pseudoaneurysm with clear traumatic pathogenesis as an isolated injury, challenging the assumption that AVP formation requires high kinetic energy and is associated with multiple injuries.

The pathogenesis of thoracic aortic injury following deceleration mechanisms and shearing forces is better understood. Similarly, for AVPs, shearing forces at the junction of the mobile azygos arch and the relatively fixed ascending portion of the vein near the vertebral column may contribute to pseudoaneurysm formation. Further, pre-existing vasculopathy or endothelial damage could predispose to pseudoaneurysm formation due to a previously weakened azygos vein.

The reported management of AVAs is multifaceted, ranging from expectant management to embolization to surgical resection via either video-assisted thoracoscopy or thoracotomy, depending on various clinical factors and perceived risks of complications [12,14]. Reported indications for intervention include rupture, large size with compressive symptoms, thrombus within the aneurysm, and patient choice [2,15,16]. Most reported cases requiring intervention had aneurysms 3-6 cm in diameter, much larger than the 1.4 cm pseudoaneurysm in this case [7,16]. However, larger AVAs have also been successfully managed with observation [10,11]. Interestingly, McDermott et al. reported a case of traumatic azygos rupture with associated hemothorax that was successfully managed by observation alone [9].

This case demonstrates complete resolution of a pseudoaneurysm at an eight-month follow-up, suggesting that spontaneous healing is possible, which is likely aided by the low-pressure nature of the venous system. Spontaneous healing of pseudoaneurysms may be more favorable than in true AVAs, as seen in a report by Mohammad et al., where a true idiopathic AVA showed no size change on a one-year follow-up CT [11]. Nonetheless, the natural history of AVAs and AVPs remains uncertain.

## Conclusions

We present a case of nonoperatively managed AVP secondary to blunt chest trauma. This rare condition was even more atypical, as it was an isolated injury. Pre-injury imaging validated the trauma-induced formation of this AVP. Our case supports the potential for non-invasive management with observation for smaller, uncomplicated pseudoaneurysms. The nuanced decision-making process in AVA/AVP management reflects the need for balancing intervention risks with the uncertain natural course of the condition.
